# Workload, mental health and burnout indicators among female physicians

**DOI:** 10.1186/s12960-016-0108-9

**Published:** 2016-04-01

**Authors:** Zsuzsa Győrffy, Diana Dweik, Edmond Girasek

**Affiliations:** Institute of Behavioural Sciences, Semmelweis University, Nagyvárad square 4, Budapest, H-1089 Hungary; Department of Obstetrics and Gynecology, University of Szeged, Semmelweis st. 1, Szeged, H-6725 Hungary; Health Services Management Training Centre, Semmelweis University, Kútvölgyi st 2, Budapest, H-1125 Hungary

**Keywords:** Female physicians, Mental health, Burnout, Sleep disorders, Workload

## Abstract

**Background:**

Female doctors in Hungary have worse indicators of physical and mental health compared with other professional women. We aimed to cast light on possible indicators of mental health, workload, and burnout of female physicians.

**Methods:**

Two time-points (T) were compared, in 2003 (T1 *n* = 408) and 2013 (T2 *n* = 2414), based on two nationally representative surveys of female doctors, and comparison made with data from other professional control groups. Independent samples *t* test or chi-squared test was used both for the two time-point comparison and the comparison between the index and the control groups. The background factors of sleep disorders and burnout were assessed by binary logistic regression analysis.

**Results:**

No significant differences in the rates of depressive symptoms and suicidal thoughts and attempts were detected between the 2003 and 2013 cohorts, but the prevalence of sleep disorders increased. The workload increased, and there was less job satisfaction in 2013 than in 2003, coupled to more stressful or difficult work-related situations. The personal accomplishment component of burnout significantly decreased in line with the declining work-related satisfaction. Compared to the professional control groups, the prevalence of depressive symptoms, suicide attempts, and sleep disorders was higher among female physicians at both time-points. The number of workplaces, frequency of work-related stressful situations, and intensive role conflict was associated with sleep disorders and decreased personal accomplishment.

**Conclusions:**

In comparison with the other professional groups, female doctors had worse mental health indicators with regard to depression, suicidal ideas, and sleep disorders both in 2003 and 2013 while within professional strata the changes seemed to be less. Increasing workload had a clear impact on sleep disorders and the personal accomplishment dimension of burnout.

## Background

Internationally, there has been a focus on the physical and mental health and on the stress burden that doctors have often to face. The issue is of considerable importance because adverse health will affect the effectiveness of health care [1].

Surveys focusing on the prevalence and severity of different psychological conditions among physicians have indicated that they can be considered as high-risk group concerning mental health. Problems of mental health affect one quarter to one fifth of the general population, but one third of doctors have been shown to suffer from at least one such condition [2, 3]. Tyssen et al.’s longitudinal survey stands out among research on physicians’ well-being. Their survey of four time-points found that Norwegian doctors were less satisfied with their lives compared with the members of a professional control group [4].

High levels of stress lead to symptoms of anxiety in almost one third of physicians and depression affects 20 percent of them. Such symptoms are more prominent among female physicians and while the life-time prevalence of depression in the general population is around 25 %, it may be up to 39 % among female doctors [5–7].

Sleep disorders are prevalent among physicians, especially among females. The prevalence among Spanish female doctors was found to be between 35 and 40 % [8, 9]. The importance of sleep disorders has been emphasized in many studies which have shown that changing work schedules, night shifts, and the consecutive fragmented sleep could have a severe cognitive and emotional impact on the performance of physicians on the day after a shift. Changing work schedules and sleep deprivation have been suggested as triggers of subsequent sleep disorders [10], and as potential predictors of chronic diseases such as hypertension, diabetes, and cardiovascular disease [11].

There have also been publications that focused on suicidal behavior among physicians. Schernhammer et al. found in a meta-analysis that suicide was almost one and a half times more frequent among male physicians compared with the general population, while in case of female doctors the multiplier was two [12]. Suicidal ideation was also found to be more prevalent among physicians than in the general population [13, 14].

Burnout has become a leading challenge within health care in the twenty-first century [15]. Severe emotional and physical strain along with the lack of positive feed-back leads to exhaustion, cynicism, and decreased performance. Dissatisfaction with work is also an important determinant of burnout. Lee et al. in their meta-analysis emphasized that the key dimension of burnout was emotional exhaustion which was found to be a first component in the development of the three dimensions of burnout. Dissatisfaction with distinct aspects of work had the greatest impact on emotional exhaustion. These aspects were autonomy, the power to make decisions, the workload, and working hours [16]. These results were confirmed by Shanefelt et al. among oncologists. They found that not even heavy emotional and physical workload led necessarily to burnout if the physicians were satisfied with their job and the work conditions [17]. Several researchers have mentioned the difficulties of balancing between work and family as important determinants of burnout [18, 19]. The mental condition of health care staff is, however, not a private issue for those concerned. Burnout can have a significant impact on the prevalence on complications and on malpractice [20, 21].

The investigation of female physicians is important. Previous studies have shown that mental vulnerability is more prevalent among female doctors than among their male counterparts. As more women have become medical doctors in recent years, this gains added significance [9, 12]. In the USA, the proportion of female university students was around 10 % in the 1960s, but has become more than 50 % by now [22]. Interestingly enough, although prognoses from the early 1990s forecasted that every third physicians would be female by 2010, it has become clear that almost half of medical doctors are female and 60 % of medical students. [23–25].

Nevertheless, there is a difference between the Hungarian and the European or North American tendencies. The proportion of female medical students and doctors has been relatively higher in Hungarian health care and in neighboring countries since the 1950s [26, 27]. In contrast with their North American and Western European counterparts, Hungarian female physicians have been working full-time and their workload has been equal to that of their male colleagues for almost seven decades.

This situation has inspired several Hungarian investigations. Győrffy et al. conducted a survey of 408 physicians and the results were compared with the professional control group in the Hungarostudy 2002 database [28]. Symptoms of depression, suicidal ideation, and sleep disorders were more frequent among both male and female physicians compared with other professionals. Reproductive disorders and chronic diseases such as hypertension, cardiovascular, musculoskeletal, gastrointestinal, and gynecological diseases were more frequent among female physicians than in the professional control group. Moreover, certain chronic diseases developed at an earlier age and in clusters. On investigating the background factors of the diseases, contact with harmful chemical agents, increased prevalence of sleep disorders, and a medium to high level of role conflict proved to be important risk factors beyond the traditional ones [29].

In 2013, we performed a nationwide, representative survey with the aim of describing Hungarian physicians’ physical and mental health. Our objectives were to compare the results of the two representative surveys of female physicians and to determine the degree and direction of changes with time. According to our hypothesis, this two time-point study supports the notion of the “Central European paradox of female physicians” which refers to that the physical health condition of Hungarian female doctors appears to be worse both in an international and in a Hungarian professional comparison. According to the first analysis of data from 2013, somatic and reproductive health indicators of female physicians suggested that chronic diseases and reproductive disorders were more characteristic of female physicians than in the professional control sample. Trends of the disease indicators were found to be the same as in the earlier survey from 2003 [30].

Our objective in the present analysis was to compare the results of the two representative surveys of female physicians from 2003 and 2013 with special regard to mental health, workload, and work stress. We aimed to determine the degree and direction of changes and to compare the mental health indicators of female physicians with those of a professional control group.

## Methods

A quantitative, online survey of physicians and dentists working in Hungary was conducted between 9 May and 15 July, 2013. The survey was performed with the permission of the Hungarian Medical Chamber. Ethical permission was obtained from the Ethical Committee of Semmelweis University, Budapest (No: 60/2013).

A link to an anonymous self-administered questionnaire was sent to all potential participants, namely to the registered members of the Hungarian Medical Chamber with valid e-mail address (*n* = 42.342). This was followed by four reminder e-mails. Response rate was 16.18 % (*n* = 5.607). We considered a questionnaire complete in case the participant responded to at least 90 % of the questions. All international studies emphasize that surveys of physicians have lower response rates even in case of traditional, paper-based surveys in comparison with the general population [31, 32]. The response rates among physicians average about 10 % lower than studies with the general population. Studies on physician surveys have shown that lower response rates were the result of using web surveys alone compared to other survey modes [32].

The data were weighted by gender, age, and type of profession (physicians vs. dentists), according to the data on members of the Hungarian Medical Chamber. After three-dimensional weighting (gender, age, and type of profession), the distribution of data concerning the region and type of workplace were compared with the same type of data from the Hungarian Central Statistical Offices [33]. As a result of the comparison, we found no distributional differences by regions (counties) or by type of workplace (general practice, in-patient and out-patient care). Therefore, we considered our survey material representative. Due to the special nature of their profession, dentists were examined as a separate group. In the present analysis, we limited our attention to female medical doctors (*n* = 2414).

Among mental health indicators, we focused on depressive symptoms, suicidal thoughts and attempts, and different dimensions of sleep disorders. In the analysis, the following work-related variables were used: number of workplaces, working hours per day, changing work schedule (night shifts), satisfaction with work, and frequency of work-related stressful events. In addition, we analyzed indicators of role conflict and burnout.

The first representative study on Hungarian female physicians was conducted in 2003 in which we used *random starting point* systematic sampling among the registered members of the Hungarian Medical Chamber. Two thirds (62.7 %, *n* = 408) of the questionnaires completed by the sample chosen were then suitable for statistical analysis. This sample was weighted according to the age and type of workplace dimensions provided by the Hungarian Medical Chamber and the Hungarian Central Statistical Offices. Thus, our sample was considered representative with regard to age and type of workplace, which are the most important dimensions from the perspective of this survey.

### Control group

With regard to the questions related to mental health (depression, suicidal thoughts and attempts), we used control groups from the population-based surveys Hungarostudy 2002 (*n* = 12.640) and Hungarostudy 2013 (*n* = 2.000). The objectives of these representative surveys were to describe the physical and mental health condition of the Hungarian population and to investigate the association between health indicators and certain environmental, social, and economic background factors. Altogether, 12.640 and 2.000 respondents, respectively, were interviewed about their socio-demographic data, health condition, behaviors and habits, and psychological characteristics. Participants of both surveys represented the adult population in Hungary according to gender, age, and place of residence. Each questionnaire consisted of approximately 200 items, and answers were obtained via interviews and self-administered questionnaires. For the comparison with female physicians, the data of 818 and 146 professional women were used, respectively [34, 35]. Professional women mean women who graduated from college or university. The professional control groups were representative in both time-points; thus, we compared two samples that were adjusted for gender, educational level, and age.

### Measurement of mental disorders

The following tools were used for the assessment of mental health factors:

*Sleep disorders* were assessed by the shortened Hungarian version of the Athens Insomnia Scale (AIS) [36, 37]. The original scale consists of 8 items, e.g., such as “Have you had problems with your sleep during the last month that occurred at least three times per week?” or “Do you have difficulties falling asleep?” or “Do you wake up in the middle of the night?” or “Do you wake up too early in the morning?”; total sleep duration (0: no problem, 1: minor problem, 2: considerable problem, 3: serious problem). The cut-off score of 10 in epidemiological surveys is adequate. In our sample, the Cronbach alpha value of the Hungarian version of AIS is 0.874.

We assessed *depression* by the shortened version of Beck Depression Inventory (BDI) [38]. Converting the total score of the shortened Beck Depression Inventory to the original BDI, four grades of depression can be distinguished (total score/9 × 21): 0–9: indicates no depressive symptoms; 10–18: mild depressive symptoms; 19–26: moderate depressive symptoms; above 26: severe depressive symptoms. In our study, the Cronbach alpha value of BDI was 0.86.

In the survey, we used the modified question of Paykel’s “Suicidal ideation and suicide attempts” questionnaire [39] to assess *suicidal thoughts*: “Have you ever been preoccupied with suicidal thoughts?” The answers (1. no suicidal thoughts, 2. suicidal thoughts in the past year, 3. suicidal thoughts in the past 5 years, 4. suicidal thoughts for more than 5 years) were dichotomized as follows: 1. never have had suicidal thoughts, 2. have had suicidal thoughts.

To assess suicide attempts, we used the following question: “Have you ever attempted suicide?” Answers (1. never, 2. suicide attempt in the past year, 3. suicide attempt in the past five years, 4. suicide attempt for more than five years) were dichotomized as follows: 1. have never attempted suicide, 2. have attempted suicide.

Burnout was measured using the Hungarian version of Maslach Burnout Inventory-Human Services Survey (MBI) [40, 41]. This questionnaire of 22 items has three subscales for the measurement of the three different dimensions of burnout: emotional exhaustion subscale (EE), depersonalization subscale (DP), and personal accomplishment subscale (PA). EE consists of nine items and assesses the feelings of being overextended and exhausted in one’s workplace (e.g., “I feel depressed at work”). DP has five items and measures an unfeeling and impersonal response toward recipients of one’s service, care treatment, or instruction (e.g., “I don’t really care what happens to some recipients”). PA consists of eight questions assessing feelings of competence and success in one’s work (e.g., “I have accomplished many worthwhile things in this job”).

The items were rated on a seven-point Likert scale according to the prevalence of certain work-related feelings (from 0 = “never” to 6 = “every day”). By the trisection of the total score reached on each subscale, three levels of burnout could be detected (1. mild, 2. moderate, 3. severe). Among physicians, severe burnout was characterized by the following cut-off scores: EE >27, DP >10, and PA <33. In the present analysis, Cronbach alpha score of the EE, DP, and PA was 0.909, 0.767, and 0.818, respectively.

One questions represented a role conflict [42, 43]: “How often do you feel irritated or dissatisfied because of the impression that you cannot balance between your workplace, family, household, or partnership engagements?”, which the respondents answered on a five-point Likert scale (1. never, 2. rarely, 3. sometimes, 4. often, 5. very often). This variable was dichotomized in certain parts of the analysis: 1. never, rarely or sometimes experience role conflict, 2. often or very often experience role conflict.

We aimed to examine the frequency of work-related stressful events by the question: “How often do you experience stressful situations during work?” This was answered by the respondents on a five-point Likert scale (1. not typical at all, 5. absolutely typical). As the result of dichotomization, two categories emerged: 1. not typical at all, 2. typical/absolutely typical.

The degree of subjective workload was assessed by the following question: “How often do you feel overloaded?” which was answered by the respondents on a five-point Likert scale (1. not typical at all, 5. absolutely typical). As the result of dichotomization, two categories emerged: 1. not typical at all, 2. typical/absolutely typical.

We wanted to detect changing work schedule, with primary focus on shifts by the questions: “Do you work in shifts? How is it scheduled?” The possible answers were dichotomized into two values: 1. do not work in shifts, 2. work in shifts. In the second case, we queried the number of hours being on duty.

The number of workplaces was grouped as follows: 1. only one workplace, 2. two or more workplaces. In case of weekly working hours, we counted the average daily working hours and dichotomized the results: 1. less than 8 h, 2. more than 8 h.

### Statistical analysis

During the descriptive analysis, we calculated frequency, mean values, and standard deviation. Deviations of percentages are indicated also. Depending on the type of variables, independent samples *t* test or chi-squared test was applied. Binary logistic regression analysis was performed to assess the correlation between work-related background factors and the items that became more prevalent among female physicians over a decade (sleep disorders, decreased personal accomplishment). Dependent variables were dichotomized variables of AIS and PA (moderate and severe). Independent variables were age, number of workplaces, amount of working hours, night shifts, work-related satisfaction, work-related stressful situations, and indicators of role conflict.

In the present analysis, we refer to the ratio of valid answers. For the statistical analysis, we used SPSS 15.0.

## Results

### Demographic data of female physicians from the 2003 survey

The average age of the participants was 45.1 years. Two thirds (40 %) of female physicians worked in the capital and 25 % in larger and 24 % in smaller cities, whereas 11 % worked in villages.

The majority of the respondents (71.7 %) were married or cohabiting. With regard to marital status, 8.9 % were single, 4.2 % cohabiting, 67.5 % married, 10.7 % divorced, and 5 % widowed. The 17.8 % of the respondents were childless, whereas 26.7 % of the respondents had one, 46.2 % had two, and 9.3 % had three or more children. Distribution of the participants’ workplace was the following: 36.4 % worked in in-patient care, 20.9 % in out-patient care, 26.9 % in general practice, and 15.8 % somewhere else (e.g., non-governmental organization, private practice, multinational company, administration).

### Demographic data of female physicians from the 2013 survey

The average age of the participants was 49.5 years. One third (33 %) of female physicians worked in the capital and 30 % in larger and 30 % in smaller cities, whereas 5 % worked in villages.

With regard to marital status, 10.5 % were single, 13.9 % cohabiting, 59.4 % married, 11.3 % divorced, and 5.4 % widowed. The 23.8 % of the respondents were childless, whereas 21.4 % of the respondents had one, 40.1 % had two, and 14.7 % had three or more children. Distribution of the participants’ workplace was the following: 35 % worked in in-patient care, 24.6 % in out-patient care, 26.1 % in general practice, and 14.3 % somewhere else (e.g., non-governmental organization).

### The professional control group in 2003 (*n* = 818)

The control group was adjusted to the sample of female physicians by age; the mean values and the frequencies (%) are the same in the case of this dimension. Almost one fifth (11.5 %) of the respondents were single, 70 % were cohabiting and married, 14.1 % were divorced, and 4.4 % were widowed. Of them, 17.8 % were childless, 26.7 % had one, 46.2 % had two, and 9.3 % had three or more children.

### The professional control group in 2013

The professional control group (women with a college or university degree) consisted of 146 women. The average age of professional women was 48.1 years. Almost one fifth (18.1 %) of the respondents were single, 11.1 % were cohabiting, 48 % were married, 18.1 % were divorced, and 4.7 %were widowed. Almost one third (31.9 %) of them were childless, 29.3 % had one, 33.8 % had two, and 4.9 % had three or more children.

### Mental health indicators

Female physicians reported mild, moderate, and severe depressive symptoms more frequently in 2013 than in 2003; however, this was not a statistically significant difference (Table 1). Female physicians were more affected by mild depressive symptoms than the professional control group in both time-points (*p* < 0.001 in 2003 and *p* < 0.002 in 2013).

**Table 1:** BDI scores of female physicians and control group in 2003 (T1) and 2013 (T2) % (*n*)

BDI	T1 (*n* = 406)	Control group 2003	*p*	T2 (*n* = 3039)	Control group 2013 (*n* = 128)	*p*
Mild	14.2	12.3	0.283	16.8	12.8	<0.001
Moderate	4.1	2.5	0.004	4.6	3.8	0.789
Severe	1.2	1.2	0.348	1.2	2.1	0.412

### Suicidal ideation and suicide attempts

In 2003, 20.3 % of female physicians reported suicidal thoughts, while in 2013 this ratio was 20.2 %. We did not find significant differences from 2003 to 2013.

In comparison with the general population, significantly more female physicians reported suicidal thoughts in 2013 (9.6 vs. 20.3 %, respectively; *p* < 0.001). In 2003, 20.3 % of female physicians reported suicidal thoughts, whereas it was 10.8 % in the control group (*p* < 0.000; data not shown). In case of suicide attempts, we did not find differences between the two surveys: the prevalence among female physicians was 2.4 % in 2003, whereas in 2013 it was 2.2 %. Suicide attempts were more prevalent in the general population (2.4 vs. 3.5 % in 2013 and 2.4 vs. 2.9 % in 2003), but the difference was not significant.

### Sleep disorders

The quantity of sleep serves as an important indicator of workload. In the comparison of the workday and weekend hours of sleep of female physicians, we did not find significant difference from 2003 to 2013 (Tables 2 and 3). According to Athens Insomnia Scale, 32.5 % (*n* = 132) of female physicians in 2003, while 39.2 % (*n* = 1162) of them in 2013 suffered from any kind of sleep disorder (*p* < 0.023). In 2003, 18 % of the professional control group suffered from sleep disorders, according to AIS scale (no related data from 2013; Tables 2 and 3).

**Table 2:** Quantity of sleep on workdays and on weekend days among female physicians in 2003 (T1) and in 2013 (T2) hour (SD)

Quantity of sleep	T1	T2
Average hours of sleep on workdays (hours and SD)	6.759 (SD = 0.898)	6.715 (SD = 1.189
Average hours of sleep on weekend days (hours and SD)	8.07 (SD = 1.48)	7.99 (SD = 1.399)

**Table 3:** Distribution of sleep disorders % (N) among female physicians in 2003 (T1) and in 2013 (T2)

Sleep disorders	T1	T2	*p*
Sleep disorder that was present at least three times a week in the past month	32.5 (132)	39.2(1162)	<0.004
Problems with sleep induction	6.8 (28)	8.1( 243)	<0.018
Interrupted sleep	19.2 (78)	14.7 (446)	<0.003
Too early awakening	6.1 (25)	10.4 (315)	<0.004
Inadequate duration of sleep	5.1 (21)	11 (335)	<0.001
Inadequate quality of sleep (independently of quantity)	8 (33)	12.4 (254)	<0.004
Sleep disorder that interferes with physical and mental daytime activity	49 (200)	65.7	<0.001
Sleep disorder resulting in exhaustion, fatigue	32.5 (132)	43.3	<0.001

### Work-related indicators of female physicians

The most important work-related characteristics of the female physicians are shown in Table 4. In this analysis, we used data from those female physicians who actively worked at the time when the surveys took place. Significant increases were found with regard to the average number of workplaces from 2003 to 2013: i.e., 1.3 (SD = 0.7160) vs. 2.4 (SD = 0.984), respectively (*p* < 0.032). In 2003, 57.8 % of the sample worked more than 8 h per day on average, whereas this ratio was 82.4 % in 2013 (*p* < 0.000). In the 2003 sample, 45.4 % of the respondents worked in shifts, while in 2013, it was 48 %. We did not find significant difference from 2003 to 2013. Almost one third (30 %) of female physicians were satisfied or very satisfied with their work in 2003, whereas the rate of these respondents was 21.3 % in 2013 (*p* < 0.000). In 2003, 55.3 % of female physicians reported that stressful situations emerged often or very often during work, whereas this number was 65.9 % in 2013 (*p* < 0.000). In 2003, 42.8 % of female physicians reported frequent or very frequent feelings related to role conflict. In 2013, this number was 45.3 %. We did not find significant difference from 2003 to 2013.

**Table 4:** Work-related indicators of female physicians in T1 (2003) and T2 (2013)

Work-related indicators	T1 (%)	T2 (%)	*p*
Number of workplaces (average)	1.3	2.48	0.032
Daily working hours (more than 8 h)	57.8	82.4	0.001
Shift work	45.4	48	0.789
Satisfied or very satisfied with work	30	21.3	0.001
Stressful situations	55.3	65.9	0.001
Frequent or very frequent role conflict	42.8	45.3	0.569

### Burnout

We examined the changing degree of different dimensions of burnout among female physicians. The average score on the EE was 19.069 (SD = 8.72) in T1 and 19.23 (SD = 11.99) in T2, on the DP 4.3 (SD = 5.45) in T1 and 5.6 (SD = 5.64) in T2, and on the PA 34.84 (SD = 9.55) in T1 and in T2 34.6 (SD = 8.947), *p* < 0.000). The distribution of mild, moderate, and severe scores reached on each subscale of MBI is shown in Table 5. There was a remarkable increase in the moderate level of depersonalization, and a significant decrease in the moderate and severe level of personal accomplishment from 2003 (T1) to 2013 (T2).

**Table 5:** Burnout of female physicians in T1 (2003) and in (2013) T2 (%)

Dimensions of burnout	Mild (%)	Moderate (%)	Severe (%)
Emotional exhaustion			
T1	50	30.4	19.6
T2	48.6	30.2	21
*p*	0.256	0.196	0.423
Depersonalization			
T1	60.7	28.1	11.2
T2	62.3	23.7	14
*p*	0.569	<0.001	0.123
Personal accomplishment			
T1	65.3	19.2	15.5
T2	33.7	27.8	38.5
*p*	<0.001	<0.001	<0.001

### Determinants of sleep disorders and decreased personal accomplishment, according to bi- and multivariate analysis

We examined the role of work-related factors in the background of increased AIS scores and decreased personal accomplishment from 2003 to 2013. We also built up a multivariate model to explain these changes. According to the bivariate analysis, high AIS scores were associated with the number of workplaces, the amount of working hours, night shifts, work-related stressful situations, and the intensity of role conflict. Moderate and severe level of decreased personal accomplishment was associated with the amount of working hours, shift work, work-related stressful situations, and the intensity of role conflict. In the background of both sleep disorders and decreased personal accomplishment, more than one workplace, frequent work-related stressful situations, and intensive role conflict were detected by multivariate analysis (Table 6).

**Table 6:** Work-related determinants of AIS and moderate to severe decrease of personal accomplishment, according to multivariate analysis

Variables	AIS OR and CI 95 % in multivariate analysis	*p*	PA OR and CI 95 % in multivariate analysis	*p*
More than one workplaces	1.354 (1.112–1.648)	0.02	1.246 (1.094–1.420)	0.03
>8 h of work/day	1.087 (0.756–1.558)	0.69	1.000 (0.669–1.528)	0.96
Night shifts	0.742 (0.523–1.058)	0.74	0.965 (0.678–1.384)	0.78
Low level of work-related satisfaction	1.000 (0.789–1.593)	0.61	1.142 (0.781–1.648)	0.74
Frequent work-related stressful situations	1.276 (1.056–1.542)	0.01	1.197 (1.063–1.348)	0.04
Frequent role conflicts	1.488 (1.242–1.783)	0.01	1.748 (1.541–1.983)	0.01

## Discussion

It is a paradigm in health sociology that health status has a strong association with the educational level; therefore, highly educated women are less likely to suffer from somatic diseases [44]. However, our previous and present surveys have shown that significant differences in health status can be found even among groups of highly educated people. According to our results, female physicians had worse subjective and objective health indicators in comparison with the professional control group. These results are equivocal with the findings of domestic research groups. On the other hand, the significant deviation from the international trends is obvious [45, 46]. International studies of somatic health emphasize that doctors are more informed about the methods of prevention and health-preserving techniques; moreover, they have better chances in the early perception and treatment of certain conditions. On the other hand, the emotional and physical burden that characterizes medical profession can lead to the over-representation of health problems among physicians [47–49]. Nevertheless, we did not find similarly negative trends of somatic health indicators among female physicians working in foreign countries; therefore, we consider this as a rather unique Hungarian phenomenon.

In this study, we surveyed representative samples of Hungarian female physicians to describe their mental health, workload, and burnout indicators in two time-points. The design of a two time-point survey enabled the detection of the degree and direction of changes occurring with time. According to our results, significant changes were detected between the two time-points of data collection, especially in relation with the amount of workload and work stress. On the one hand, the frequency of depressive symptoms and suicidal thoughts did not change markedly. On the other hand, not only the overall prevalence but also that of each item of sleep disorders increased. Also quantity of workload showed an increase: the amount of daily working hours and number of workplaces rose in comparison with the data of the 2003 survey. In line with this, female doctors reported less overall work-related satisfaction and more burdensome and stressful situations in 2013. The intensity of role conflict slightly enhanced, whereas personal accomplishment dimension of burnout decreased markedly in line with decreasing work-related satisfaction. Bi- and multivariate models showed that heavy workload (number of workplaces, frequency of work-related stressful situations, intensity of role conflict) was associated with higher AIS scores and moderate to severe decrease of personal accomplishment.

Related studies found the prevalence of depression to be 7–56 % among physicians, which was two times more than that of the general population [50]. Researchers in the HOUPE study showed that the prevalence of suicidal thoughts among Swedish and Italian physicians was around 12 % [51]. Studies focusing on physicians affected by insomnia estimated the rate of this condition to be 35 % [52].

In the survey of Hungarian female physicians in 2003, a wide range of chronic diseases were more prevalent among physicians compared with the professional control group. This phenomenon was described as the “health paradox of female physicians”. The finding that the prevalence of depression and suicidal thoughts and attempts did not change over a decade needs to be emphasized. However, depression and EP did not change from T1 to T2, while AIS, PA, and the amount of workload did. This might be explained by the following: emotional exhaustion and depression can be considered as a response to emotional strain, whereas sleep disorders and decreased personal accomplishment are more likely to be due to the increased amount of workload. Therefore, it is likely that it is not work-related emotional strain that increased from T1 to T2 change with time, but increasing workload led to more sleep disorders and decreased personal accomplishment, as it was shown by our survey.

A considerable change in the amount of workload and work-related stress was detected. In the background of the increasing workload, the role of the headcount and distribution of different physician groups can be assumed. Migration, ageing, and retirement of doctors are important contributors to the human resource crisis of the health sector. Similarly to the worldwide known phenomenon, migration of physicians is one of the most urgent challenges of health policies. According to the latest data, altogether 948 Hungarian physicians applied for the official work permit needed for working abroad in 2014 [53]. It was shown by a Hungarian study in 2011, that the most important motivations for working abroad were based on better salary, quality of life, perspectives of Hungarian health care, work conditions, appreciation of the community, and professional perspectives in one’s field of specialization [54]. Ageing of the medical community also contributes to the ever expanding workload. At present, the most populous age group of practicing Hungarian physicians is between 55 and 60 years. It can be expected that the retirement of such a large cohort would lead to serious shortage in human resources [55]. Especially the ageing of general practitioners and the unresolved succession have been one of the major problems of Hungarian health care for many years. [56]. Underfunding of the health sector is the third important contributor to increased workload. Low salaries given to physicians inevitably lead to having more workplaces simultaneously or to taking on extra night shifts. Thus, increasing workload might lead to a more explicit role conflict and to decreased effectiveness, the latter of which was most apparently present in the personal accomplishment dimension of burnout, according to our results. Previous studies highlighted that lowered personal accomplishment is in correlation with the loss of control over one’s work, which is a substantial issue of the functioning of Hungarian health care [57]. Nevertheless, it is important to note that the basic dimensions of burnout are depersonalization and emotional exhaustion, and several studies do not consider the personal accomplishment subscale a part of burnout assessment [58]. Personal accomplishment means feeling of competence and successful achievement in one’s work with other people [43]. Still, previous domestic studies found significant decrease in this dimension of burnout; therefore, dealing with this issue is inevitable. Moreover, it is the female population, including female doctors, who are primarily affected by household-related workload. According to our survey from 2013, there was no significant difference in the average weekly working hours between male and female physicians, whereas female doctors spent on average much more hours on housewifery than their male counterparts. In line with this, female doctors reported significantly less leisure time than their male colleagues [59].

Besides the outstanding stress burden [60] imposed on medical professionals, several domestic peculiarities contribute to the paradox of Hungarian female physicians. The mentally and physically burdensome work of Hungarian female doctors is further aggravated by phenomena that have recently characterized the Hungarian labor market: ageing and migration of physicians and the general underfunding of the health care sector. Furthermore, there is a widespread overload of roles. Virtually, there is no division of labor in the partner relationships, which results in a significant extra burden due to the “invisible” workload imposed on women (housewifery, caring for children, and the elderly). Also, there are hardly any part-time job options. It is likely that due to all the abovementioned factors Hungarian female physicians are more vulnerable both somatically and mentally compared with other professional women.

Representative samples of female physicians and the comparison with representative, professional control groups compose the primary strength of this research. Furthermore, we used validated tools when examining mental health factors. Among the weaknesses, a few variables (work-related satisfaction, stressful situations, and role conflict) should be mentioned that were assessed by only one item. We also stress the need for further research aimed at disclosing the background factors of mental health and its association with workload. Further limitation is that the methods of data collection in the 2003 and 2013 quantitative surveys differed (paper-based vs. online, respectively). Furthermore, respondents in the two surveys were not the same people. We also have to mention that the exact distribution of professions in the professional group is unknown. A relatively low response rate is a further limitation; however, multi-dimensional weighting was aimed to get respondent groups that were more representative.

## Conclusions

In this study, the paradox of Hungarian female physicians gained further confirmation. In comparison with the general population, female doctors lagged behind with regard to indicators of mental health (depression, suicidal ideation) both in 2003 and 2013. There was a remarkable increase in the prevalence of sleep disorders and lowered personal accomplishment by 2013. These findings were found to be associated with increasing workload.

### Ethical approval

The study was approved by the Ethics Committee of the Semmelweis University, Budapest (ref. number: 60/2013). Our study design included anonymous and voluntary answers to online questionnaires from physicians, but no invasive sampling methods or other similar techniques were applied; thus, there was no need for an informal consent from participants. According to the Ethics Committee of the Semmelweis University’s statement, “no informal consent is necessary when conducting researches in the field of social sciences”. Our research was conducted in full accordance with the World Medical Association Declaration of Helsinki.

## Abbreviations

AIS: Athens Insomnia Scale
BDI: Beck Depression Inventory
DP: depersonalization subscale
EE: emotional exhaustion subscale
MBI: Maslach Burnout Inventory
OR: odds ratio
PA: personal accomplishment subscale
SD: standard deviation
